# Supplementary material for: School counselor advocacy for gender minority students
Source: PLoS One. 2021 Mar 17;16(3):e0248022. doi: 10.1371/journal.pone.0248022 (PMC7968657; doi:10.1371/journal.pone.0248022)
Supplement: S1 File — (DOCX) [file pone.0248022.s001.docx]

**Demographic Items for School Counselor Gender Minority Advocacy Competence Study**

1. What is your age? (select one)

___ 20 to 29

___ 30 to 39

___ 40 to 49

___ 50 to 59

___ 60 to 69

2. What is your gender? (select one)

___ Male

___ Female

___ Intersex

___ Transgender

___ Other*

*If Other, please fill in the blank. ________________________

3. Which of the following best describes your race/ethnicity? (check as many as apply)

___ African American

___ European American

___ Hispanic

___ Multiracial

___ Other*

*If other, please fill in the blank. _________________________

4. What is your sexual orientation? (select one)

___ Exclusively Heterosexual

___ Mostly Heterosexual

___ Mostly Lesbian/Gay

___ Exclusively Lesbian/Gay

___ Other*

*If Other, please fill in the blank. _________________________

5. What of the following best represents the school level that you work at? (select one)

___ Kindergarten School Level

___ Elementary School Level

___ Middle School Level

___ Middle and High School Levels

___ All Levels

___ Other*

*If Other, please fill in the blank. _________________________
