# Supplementary material for: School counselor advocacy for gender minority students
Source: PLoS One. 2021 Mar 17;16(3):e0248022. doi: 10.1371/journal.pone.0248022 (PMC7968657; doi:10.1371/journal.pone.0248022)
Supplement: Attachment — Submitted filename: PLOS one review.docx [file pone.0248022.s002.docx]

The article reports on survey data with a large sample size regarding an important topic. However, there are a number of places where the presentation of information needs clarifying prior to publication. Here are some suggestions:

1. Fully explain the notion of transgender intersex advocacy competency. Describe the components of “advocacy competency”? Also, outline why you are conflating transgender and intersex in the research and the scale. It is possible to be a transgender advocate but not an intersex one, or to have intersex competency but not transgender competency. Also, at the very end you begin to discuss the omission of gender queer from your survey. If you want to name that genderqueer was not included in your survey or scales then it might be better if this omission is explained and addressed at the beginning of the paper. The omission of intersex from your demographic form should also be mentioned (for example, address what impact, if any, leaving gender open-ended had on people’s comfort or discomfort in disclosing an intersex identity?).
2. All scales utilized in the study should be more clearly explained. Who developed them, where have they been used, how many questions, utilizing what kinds of response scales, and what are their psychometric properties?
3. The constant use of acronyms for numerous concepts including TI, CBAs, SCTIACS, GICCS, ICCS, IBT, TPB is distracting for the reader. It may be appropriate to use full names in places, especially for the theories.
4. In the review of literature, the author mentions 3 studies but no context is provided for these studies. The studies refer to the school setting but no data is provided re the hostile environment encountered in schools and the theoretical foundation for that hostility (e.g. heteronormativity and cisnormativity). In addition, more details about the studies, for example sample size, would be useful to assess their credibility.
5. It would be helpful to define and describe TPB. It would also be helpful to discuss whether IBT was developed in relation to TBP or simply as an alternative, in either case it would be good to know what the limitations were with TPB.
6. It would be helpful to discuss the source of the sample, the mailing lists, etc. under the “Participants and Methods” section. Also, discuss whether responses were geographically split between rural and urban, east coast, west coast, or middle of the country, etc.
7. There is reference to imputing missing data and that “most of the data were missing completely at random”, but the amount of missing data is important to know. It is also important to know which data was missing “based on sexual orientation”.
8. When reporting results there are multiple references to “Large positive relationships” for the correlation data. I believe these should be reported based on strength rather than size, so as “strong” rather than “large”.
9. The sample consists of 1190 participants and only 5 identified as transgender. I have serious concerns as to whether that cell size is sufficient to determine statistical significance without encountering error.
10. It would be helpful to describe exactly what are the components of TI competence and to discuss specifically what skills are needed and what resources are available.
11. On page 17 line 366 the author suggests that since the majority of respondents were cisgender, white, females they should be the target of efforts to develop training efforts. Such a statement further marginalizes the voices of BIPOC. Instead, I would interrogate your sampling strategies and geographic distribution of the survey to see why your sample skewed towards this demographic, and whether it is reflective of the national body.

Definitely an important topic for study and a large (though skewed) sample.
