# Supplementary material for: School counselor advocacy for gender minority students
Source: PLoS One. 2021 Mar 17;16(3):e0248022. doi: 10.1371/journal.pone.0248022 (PMC7968657; doi:10.1371/journal.pone.0248022)
Supplement: Attachment — Submitted filename: Response to Reviewers.docx [file pone.0248022.s004.docx]

Mercy College

Mahoney Hall B01

Social and Behavioral Sciences - Counseling

555 Broadway

Dobbs Ferry, NY 10522

H. Jonathon Rendina, PhD, MPH
Academic Editor
Public Library of Science (PLOS) ONE

1160 Battery St.

Koshland Building East, Suite 225

San Francisco, CA 94111 USA

February 5, 2021

Dear Dr. Rendina:

I am pleased to re-submit the manuscript entitled, “School Counselor Advocacy for Gender Minority Students” for potential publication in the *PLOS ONE* special issue on health and health care in gender diverse communities. The original manuscript was submitted to Drs. Sevelius, Scheim, and Radix. The aim of this study considering Identity Behavioral Theory was to assess the school counselor role in advocating for gender minority students (transgender and intersex students) using competency-based assessments.

A national sample of school counselor participants (*N* = 1,191) completed the School Counselor Transgender Intersex Advocacy Competence Scale, the Intersex Counselor Competence Scale, the Gender Identity Counselor Competence Scale, and a demographic form. The findings of our study suggest that counselor educators should teach current and future school counselors about how they can become more effective at advocating for gender minority students at any age by self-evaluating one’s attitudes, identities, knowledge, and skills related to this advocacy area.

I have addressed the requested the edits below:

| **Edit Comment** | **Response** |
| --- | --- |
| The first paragraph still does not explain what makes the transgender and intersex experiences "more similar" –  expanding here would help when you make a statement about there being some differences (again not specified) between the two groups on line 73.  I would focus on any important distinctions that readers should be aware of as they review your findings. | Added more information at the beginning of paragraph 3 to explain similarities and difference regarding transgender vs. intersex experiences: “Examining counselor advocacy for transgender and intersex youth is warranted because the needs of these youth appear to overlap.(2,3) The two groups experience a wide array of feelings; face challenges (e.g., being bullied); and cope with identity development.(2, 3) This is also the case for gender non-binary students.”  I also edited point 1 to make it more clear and related to findings: “We do not know how counselor competence to provide services to transgender students relates to counselor competence to provide services to intersex students. A review of the research literature indicates that no studies have empirically examined these advocacy areas together. This study appears to be the first to do so.” |
| Also, in the introduction, line 58 the definition of nonbinary is out of place here and does not relate to the subsequent sentence even though that starts with "Additionally, ..." (line 60). I would move the defining up and add a definition for trans and intersex, which are not yet defined. | I moved text up from the prior manuscript to form a new paragraph one. Then, I define each of the gender minority groups in paragraph two. New text in paragraph two reads: “In this study gender minority youth comprise those who identify as transgender and intersex. Scholars estimate 150,000 adolescents in the United States identify as transgender.(4) Transgender youth experience incongruent feelings between birth sex and gender identify.(5) Intersex individuals account for one to two percent of the population.(6) Intersex youth possess a normal variation in hormone levels or chromosomes and may experience differences in body characteristics.(7) Some will undergo genital surgery.(8) Some transgender and intersex youth also identify as gender non-binary. Gender non-binary youth, also referred to as genderqueer youth, possess non-dichotomous gender identities that are neither male nor female; the identities of gender non-binary youth may be fluid or fixed, or exist somewhere between female and male (e.g., neutral).(9,10)” |
| Page 5 line 100 the contents of the parentheses are incomplete. | I addressed this edit, and it now reads as follows: “For example, regarding the latter, school counselors should call for and run gender minority and ally groups and promote policies to assist gender minority students to transfer schools if needed (e.g., due to ongoing physical assault).(2)” |
| Additional edits/notes | **1. IBT figure has been updated as agency variable is now referred to as resilience in the model.**  **2. Additional references added to define transgender, intersex, and gender non-binary. As result, numbers in the reference list used for citation updated throughout paper and in reference list.**  **3. A preprint is now available for reference 21.**  21. Simons JD, Bahr MW, Ramdas M. Counselor Competence Gender Identity Scale: Measuring Clinical Bias, Knowledge, and Skills [Internet]. PsyArXiv; 2021. Available from: https://psyarxiv.com/d2mnu  **4. Reference 29 is in press with doi number forthcoming.**  29. Simons JD, Russell ST. Educator Interaction with Sexual Minority Youth [Internet]. EdArXiv; 2020. Available from: edarxiv.org/76fa4 |

I approve this publication as a sole author, and the publication has also been approved by responsible authorities at the institution where I presently work. I recognize that the publisher will not be held legally responsible for any claims for compensation. The manuscript has 7,689 words and is 24 pages in length (less the title, abstract, references, acknowledgments, and supporting information). The work described herein has not been submitted nor published elsewhere. It is original work and does not contain anything that is abusive, defamatory, libelous, obscene, fraudulent, or illegal. I also do not have any conflicts of interest. The manuscript adheres to Vancouver style.

If you have any questions, please contact me at jsimons1@mercy.edu or by phone at +1-212-810-0257.

Yours truly,

Jack Simons, Ph.D.
